# Learning to Be Employable Through Volunteering: A Qualitative Study on the Development of Employability Capital of Young People

**DOI:** 10.3389/fpsyg.2021.574232

**Published:** 2021-03-01

**Authors:** Maria Luisa Giancaspro, Amelia Manuti

**Affiliations:** Department of Education, Psychology, Communication, University of Bari, Bari, Italy

**Keywords:** voluntary work, informal and non-formal learning, employability capital, soft skills, career management

## Abstract

Over the last decades, consistent research showed that voluntary work could be considered as a tool for professional development and concrete employment: volunteering could be either experienced as a desire to improve career opportunities or to acquire new skills. The study aimed to investigate voluntary work as a context of informal and non-formal workplace learning and vocational guidance, useful to develop skills and abilities, namely the capital of personal and social resources, that could promote future employability. Participants were 38 young volunteers who experienced the Universal Civil Service, a national Italian program addressed to young people aged up to 28 years, giving them both the opportunity to engage in social activities useful for the community and have the first contact with a working context. In line with the objectives of the study, participants were invited to describe their volunteering experience in a diary, highlighting if and to what extent this context contributed to enhancing their employability capital, namely the asset of skills, knowledge, and networks acquired, that they could transfer to a future professional domain. The narrative data collected were examined through diatextual analysis, a specific address of discourse analysis designed to catch the relationship between enunciators, text, and context of the talk. This qualitative analysis allowed us to investigate the meanings young people attributed to these activities. In light of these results, the paper contributed to investigate volunteers’ perceptions about the conditions that could best foster this specific kind of workplace informal and non-formal learning and at proposing a qualitative perspective on the analysis of the employability capital they developed.

## Introduction

Within the last decades, the labor market has profoundly changed: a globalized economy, the fluidity of markets, a need for continuous innovation, and the increase of competitiveness are only some of the main factors that are contributing to highlight an even more central role played by human capital as a strategic – even if often intangible – factor for development and organizational success (Manuti and De Palma, 2018; Manuti and Giancaspro, 2019).

Within this frame, people can make a difference for organizations if they possess not only technical skills and professional abilities but are also smart, motivated, and eager to learn, possessing what the experts call “soft skills.” Indeed, in a fast-moving scenario, like the one outlined above, workers are called to be flexible, adaptive, must deal with the unexpected, should be able to work both independently and in a group, and should be sociable, responsible, and capable of taking initiative (Khasanzyanova, 2017). Those who possess these “soft skills” are frequently preferred over those who merely possess technical skills. This trend is also reflected in national and international policies; skills like “learning to learn,” “social and civic competence,” and a “sense of initiative and entrepreneurship” are considered key competencies in European education systems. In this vein, the non-formal and informal dimensions of learning play a primary role in the development of future generations’ career success (Schugurensky, 2000; Turner, 2006; Tynjälä, 2008; Earley, 2009; Manuti et al., 2015; Marsick and Watkins, 2015).

In this vein, volunteering could represent an important opportunity for the exploration, growth, and development of social and transferable skills, a context for informal and non-formal learning fostering the acquisition and transformation of knowledge (Callow, 2004; Souto-Otero et al., 2005) and enhancing individual employability (Nichols and Ralston, 2011; Kamerade and Ellis Paine, 2014). Recent studies confirmed that volunteering enhances both “hard” skills (business management, IT specific skills, etc.) and “soft” skills (communication, teamwork, management, and organizational skills, etc.; Peterson and Van Fleet, 2004; Cook and Jackson, 2006; Nichols and Ralston, 2011; Souto-Otero et al., 2013) thus orienting young people toward a more aware career management. However, few studies investigated this research perspective through qualitative and specifically discursive methodologies, investigating how volunteers experienced this opportunity, how they framed it, and how they consciously recognized the capital of knowledge and skills they developed through discourse. Therefore, the present study could contribute to the discussion in this important field of research also by using these “subjective” data to provide supervisors and practitioners with useful insights for the development of efficient training and career management practices that could be used to maximize the fit between volunteers and context of experience.

In view of the above, the main aim of the study was to adopt the volunteers’ perspective to understand if and to what extent a context of voluntary work – the Universal Civil Service – could grant opportunities for informal and non-formal learning useful to enrich their employability capital.

More specifically, the study was addressed to consider a specific context of voluntary work, namely young people’s participation in the Universal Civil Service Program in Italy. Second, by adopting a qualitative perspective, the study was aimed to focus on how volunteers discursively made sense of this highly relevant personal and professional experience through narratives, assuming that conscious recognition of the employability capital developed could be important to maximize this experience in the several future professional transitions.

### Volunteering as an Informal and Non-formal Context of Learning

In light of the reflections drawn above, the study considered volunteering as a context of workplace learning (Marsick and Watkins, 1990; Billett, 2001; Boud and Middleton, 2003; Ellinger and Cseh, 2007; Tynjälä, 2008, 2013; Le Clus, 2011). With the notion of workplace learning, we referred to the “many ways through which employees learn in organizations” (Jacobs and Parks, 2009, p. 134).

Accordingly, abundant research in recent years has highlighted that workplace learning is generally characterized as taking place through either formal, non-formal, or informal channels.

In their extensive literature review, Colley et al. (2003) classified learning as formal, non-formal, and informal, recognizing that the continuum of learning may range from highly formal to highly informal (Schugurensky, 2000; Hager and Halliday, 2009; Van Noy et al., 2016).

Formal learning in the workplace happens through “typically institutionally sponsored, classroom-based, and highly structured” (Marsick and Watkins, 2001, p. 25) environments. On the other hand, non-formal learning in the workplace incorporates *implicit learning*, which gives rise to tacit knowledge as well as *reactive learning*, which is spontaneous and unplanned, and *deliberative learning*, which regards systematic reflection and elaboration of past experience as fundamental for future behavior (Eraut, 2000, 2004). Finally, informal learning opposes formal learning in the workplace because it provides an environment where workers can engage in informal learning activities, thus contributing not only to the organizational effectiveness but also to the learning and development needs of individuals (Marsick and Volpe, 1999). Examples of informal learning could be found both in self-directed and in collective learning, in contexts where education or training are mostly unstructured, as, for instance, in socialization processes or in occasions for incidental transfer of learning through informal coaching and mentoring situations (Livingstone, 2006). Therefore, as many other scholars confirmed, it could be concluded that a substantial amount of workplace learning is informal and non-formal (Cheetham and Chivers, 2001; Boud and Middleton, 2003; Enos, Kehrhahn and Bell, 2003; Skule, 2004; Sambrook, 2005; Poell et al., 2006; Gola, 2009; Jurasaite-Harbison, 2009).

Moving from these assumptions and in consideration of some of the peculiarities of voluntary work featuring the experience of the Universal Civil Service Program (e.g., unstructured, informal, managed by objectives rather than by outcomes, often unreleased from supervisory control, etc.), the specific target of the present investigation, the study assumed that this context could be an informal and non-formal learning space precious to develop capital for employability.

### Volunteering and Employability

The relationship between volunteering and professional development has received considerable attention within the last decades. Empirical evidence has contributed to show that voluntary work could be considered as a tool for professional development and for concrete employment as well (Chambre, 1989; Heidrich, 1990; Hirst, 2001; Cook and Jackson, 2006; Handy and Mook, 2011). A consistent body of research confirmed that volunteering is often experienced as a desire to improve career opportunities (Katz and Rosenberg, 2005; Prouteau and Wolff, 2006; Barron and Rihova, 2011; Paine et al., 2013; Aydinli et al., 2016) and to acquire new skills (Low et al., 2007; Handy et al., 2010).

Yet, many studies showed that students involved in volunteering tended to acquire a wide range of skills applicable to different situations: decision making, leadership, creative thinking, strategic thinking, conflict resolution being the main ones (Astin and Sax, 1998; Astin et al., 1999; Cohen et al., 2014). In light of this, further contributions confirmed that volunteering has a positive impact on employability (Gay, 1998; Low et al., 2007; Paine et al., 2013) and on the development of employability skills among all age groups and professional categories (Hirst, 2001; Maranta, Sladowski, 2010; Keough, 2015).

Employability is a complex and multifaceted concept, that attracted considerable attention within the last decades especially with reference to the need of younger generations to enter the labor market and to survive in a fast-moving competitive scenario.

Among the most authoritative definitions, for the purposes of the present study, we could refer to some of the most representative, in order to focus on specific aspects of the construct. Kanter (1995) was one of the first scholars to draw attention to employability. He defined it as “[…] a person’s accumulation of human and social capital – skills, reputation, and connections – which can be invested in new opportunities that arise inside and outside the employee’s current organization” (Kanter, 1995, p. 52). More recently, Fugate et al. (2004) proposed to consider employability as a form of work-specific adaptability enabling workers to identify and realize career opportunities, facilitating the movement between jobs, both within (i.e., internal employability) and between organizations (i.e., external employability). In a similar vein, Van der Heijde and Van der Heijden (2005) conceived employability as “the continuous fulfilling, acquiring or creating of work through the optimal use of competencies” (p. 143). In this light, Lo Presti and Pluviano (2016) maintained that employability could be “a personal resource that individuals develop across their working lives aimed at increasing one’s own career success, both attaching importance and committing to making sense of past work experiences and envisioning one’s own professional future, acquiring valuable competencies and skills, improving their formal and informal career-related networks, exploring their social environment in search of opportunities and constraints to their own career pathway” (Lo Presti and Pluviano, 2016, p. 5).

Enlarging the paradigm, Peeters et al. (2019) reflected on the plurality of resources that could promote employability. In light of this, they proposed to focus on the employability capital rather than on employability as a single feature. Thus, they defined employability capital as the set of personal resources – or capital – that may impact individuals’ employability. By using this notion, Peeter and colleagues identified two key aspects that greatly contributed to developing the discussion about employability. According to the authors, there is first a capital: the personal resources through which individuals attain their goals. These resources lead to positive outcomes and can be nourished (Hobfoll, 2001). Yet, capital is the whole of knowledge, skills, and attitudes (KSA) or of social networks that could be crucial for career success (Ebya et al., 2003; Fugate et al., 2004; McArdle et al., 2007; Van der Heijden et al., 2009). Then, there is employability, which, according to this conceptualization, comes after the acknowledgment of the capital because it refers to the likelihood of obtaining and retaining a job that is strongly influenced by the ability to make one’s own capital attractive and competitive (Forrier et al., 2009).

Therefore, besides the different perspectives, what is common to the definitions presented is the evidence according to which employability is a capital; to increase this capital, one should therefore focus on investing time in activities that will increase one’s stock of human, social, and cultural resources (Smith, 2010). An effective way of increasing one’s stock of relevant capital may be by performing volunteer work. Therefore, participating in volunteer work may be a way to acquire job-related skills that are advantageous when applying for work (human capital). It may also extend social networks to include individuals with information on new job positions (social capital), and finally, volunteer work may be a way to signal one’s work ethic and social conscience to potential employers (cultural capital). These mechanisms may work parallel to increase the employability of volunteers.

Nonetheless, the premise to develop employability especially in the context of voluntary work is a focus on life-long and development, namely the ability to learn new competencies and adapt to changing circumstances (Fugate et al., 2004; Berntson et al., 2006; Van der Heijde and Van der Heijden, 2006; Fugate and Kinicki, 2008; Thijssen et al., 2008). This is what some scholars call “professional employability” or “transitional employability” (Clarke, 2008; Clarke and Patrickson, 2008), underlining the potential of transferability which is inbuilt in learning.

Moreover, to be recognized as real capital, individuals should be aware of their employability, in order to orient the asset of knowledge, skills, ability, and networks toward the attainment of congruent career goals. Accordingly, a very important dimension of employability is career identity, namely “a more or less coherent representation of diverse and diffuse experiences and aspirations” (Fugate et al., 2004, p. 19). This dimension articulates in the form of narratives, that is stories that individuals create to make sense of past, present, and future career experiences. Therefore, to access to career identity’s narratives could be useful to catch the cognitive and affective dimensions of one’s own “possible selves” (Markus and Ruvolo, 1989), organizing goals, motivations, attitudes, learning experience leading and developing a personal awareness about employability capital.

Considering this evidence, the present study adopted a qualitative methodology to study the narrative construction of the employability capital developed through informal and non-formal learning in the context of voluntary work.

## Materials and Methods

### Ethics

A complete description of the study was reviewed and approved by the Ethics Committee of the Department of Education, Psychology, Communication of the University of Bari (Ethics reference code: ET-20-11). Participants provided their written informed consent to participate in this study.

### The Context of the Research

As described above, the context of the study was the Italian Universal Civil Service, a program, regulated by the Italian Law (Law 64/2001; Dlg.40, 2017), addressed to European and non-European young people who live in Italy and want to make an experience of voluntary work. The program is mostly aimed to provide young people with occasions to develop skills that are potentially transferable to the labor market.

The mission of the program is to remove inequality and promote social integration. However, it tackles different professional fields and provides several services to the community:

- promotion of the Italian culture and support to the Italian communities abroad, enhancement of the historical, artistic and environmental heritage;
- urban redevelopment, mountain agriculture, social agriculture, and biodiversity;
- education, cultural and sport promotion, civil protection, and assistance to disadvantages categories;
- peacekeeping and non-military defense, non-violence promotion and protection of the human rights, cooperation, and development;

The young volunteers who apply for the program are tutored by an adult who is called to support them during this experience, which lasts from 8 to 12 months, facilitating their future transition to the labor market. In this vein, being a Civil Service Volunteer is a precious opportunity for workplace learning, allowing young people to grow personally and professionally. Finally, it is also recognized by the formal higher education system as it allows us to gain credits that could be spent during university experience.

### Participants and Data Collection

In light of the present research’s objectives, participants to study were 38 volunteers of the Universal Civil Service. Around 25% of the volunteers involved were men, and 75% of them were women; they were aged between 23 and 29 years (average age 25.9 – s.d. =1.8). Most of them (58%) had a bachelor’s or master’s degree and decided to undertake this experience to better understand their future aspirations. All participants were unemployed at the time of the study and provided voluntary service in the social field (assistance to the elderly, assistance to the disabled, recovery of disadvantaged children, support for families with economic problems). They were selected through a call posted on social media inviting young people who had this experience in the past 3 years and who wanted to voluntarily participate in the survey. This time extension was defined to guarantee more direct and clearer access to the memories of volunteering experience and to evaluate its effects in the short and medium term. The call specified the aims of the study and asked participants to fill in a diary page in which they were invited to narrate their experience as a volunteer of the Universal Civil Service, identifying skills and abilities they thought they have developed during this period and reporting if and to what extent has this experience contributed to define their professional goals and career choices.

In more detail, the call contained the following instructions:

“*Thinking back to the experience you made as volunteer of the Universal Civil Service, please fill in a diary page in which you try to collect your memories about it: the activities you carried out, the people you met, the difficulties you came across, and the decisions and solutions you undertake to overcome them. Please point out the skills you think you had to display to cope with this experience and those you think you have developed thanks to the Universal Civil Service. Finally, we invite you to argue if and to what extent do you think this capital of knowledge and skills and, more generally, this experience has been useful for your professional future*.”

A total of 38 texts were collected. They all contained a detailed account of volunteering experience carried out in the Universal Civil Service with special reference to the employability capital they thought they acquired through this opportunity. The conclusion of the stories was dedicated to a reflection on the usefulness of the civil service experience for the transition to the labor market. A final sheet was attached to collect basic personal information (gender, age, educational qualification, and field of intervention during volunteering).

### Data Analysis

The study adopted a qualitative perspective, being focused on the need to investigate “how” participants made sense of their experience through narratives. More specifically, the narrative data collected were analyzed by adopting a diatextual analysis (Manuti et al., 2012; Mininni et al., 2014; Mininni and Manuti, 2017). Diatextual analysis is a specific kind of discourse analysis addressed to investigate the relationship between enunciators, texts, and contexts of talk. This methodological approach suggests that the sense does not reside permanently within texts rather it goes through them (actually, the Greek prefix “dia” means “through”). Therefore, to study and to penetrate the sense that animates texts, diatextual analysis focuses on some textual and discursive traces that concretely refer to three main analytical categories: Subjectivity, Argumentation, and Modality. The acronym of these categories determines the S.A.M. model, a pragmatical support of diatextual analysis that allows us to approach texts by answering some basic questions (*Who is saying that? Why does he/she say that? How does he/she say that?*) and consequently to organize the results according to some specific patterns of sensemaking associated to the extreme variability of actors, contexts, and topics of talk.

The first question (*Who*?) aims at clarifying the discursive options that the enunciator chooses to conveys his/her identity through the text. Accordingly, with reference to the “Subjectivity” dimension, diatextual analysis allows to trace back discursive markers of agency, affect, and the enunciative strategies adopted to signal his/her position toward the discursive context and toward the interlocutors (*embrayage/debrayage* strategies) and the degree of involvement with the object of discourse.

The second question (*Why*?) points out how the enunciator argues for his/her meanings, giving voice to stakes and claims. Markers of “Argumentation” pragmatically reveal the aims and interests animating the text: these could be narrative markers (e.g., setting the scenes, characters, and models of action) or specific argumentative strategies used to legitimize one’s own position.

The third question (*How*?) focuses on the articulation of the “Modality” of discourse according to which the meaning is shaped. This diatextual category of markers is aimed to point out the stylistic and rhetorical options used to shape the relationship between the enunciator and his/her audience. Accordingly, meta-discursive markers (namely expressions of comment and reformulation), discourse genre markers (references to the typology of text and intertextual references), and opacity markers (use of rhetorical figures, frame metaphors, etc) are typical markers of modality.

The present study adopted diatextual analysis to catch how volunteers used the markers of subjectivity, argumentation, and modality to discursively construct their experience of participation in the Universal Civil Program in light of the employability capital they developed for their future professional career.

## Results

Diatextual analysis contributed to highlighting the recurrence of three main thematic networks within the narratives collected, in line with the objectives of the study. The first aim of discursive analysis was to point out how volunteers reconstructed their experience as an actual workplace learning opportunity that allowed them to experiment with their professional identity and “to learn by doing and being.” The second objective that guided diatextual analysis was to focus on the discursive description of volunteering as a privileged space to question one’s self about the future and about professional aspirations, values, and objectives in process of career self-management. Finally, diatextual analysis concentrated on volunteers’ perceptions about the precious employability capital they had the opportunity to develop through this experience.

The lens through which these objectives were met were the ones granted by the diatextual methodology, therefore focusing on the discursive traces left behind by volunteers in their discourses.

The following sections go through some extracts of the narratives in order to better argue for the results.

### Volunteering as a Learning Experience: From Doing to Being

There are many definitions of volunteering (Wilson, 2012) and the ambiguity of the concept exists because volunteering encompasses a broad range of activities (Handy et al., 2010) that are also culturally specific (Oppenheimer, 2008). Snyder and Omoto (2008) define volunteering as “freely chosen and deliberate helping activities that extend over time, are engaged in without expectation of reward or other compensation and often through formal organizations, and that are performed on behalf of causes or individuals who desire assistance” (p. 3).

The focus of this definition is on the activities that volunteers deliberately decide to undertake but volunteering cannot be understood simply as a series of activities aimed at helping others or as a complex life experience. Yet, volunteering refers to the process whereby individuals connect and engage with other persons, groups, or organizations in order to address specific community needs on an unpaid basis (McAllum, 2017). Moreover, voluntary work provides any number of intrinsic, psychic benefits. “Making the world a better place” is an important value for some people, and any time we act in accordance with our values we feel better about ourselves (Wuthnow 1991, p. 87).

In line with these assumptions, diatextual analysis of the narratives collected confirmed the strong affective and emotional involvement experienced by volunteers. Yet, one of their most evident diatextual traces is linked to the first initial in the SAM acronym, i.e., the subjectivity of discourse.

Participants’ subjectivity was discursively shaped using agency markers aimed at describing this experience as a personal journey that changed them: from the initial difficulty to measure one’s self with new contexts, people, and tasks to the sensation of complete involvement both in the activities and in the challenges connected to it. The occurrence of personal markers (“I,” “me,” “my”) underlined the personal responsibility connected to volunteering and thus the active role participants had in this experience.

1. “It took me some time to come to understand the world of those children and young people who, considering they did not know me, did not trust me, and struggled to recognize me as a figure who could help or sustain them in some way” [V1]^1^
2. “I have to admit, when the school principal included me as well, I was struck by endless anxiety and doubts: could I succeed in it? I never have had working experience with “troubled” young people, my only experience was in the field of disability. But I decided to keep the secret, I would have tried first and only later I’d have considered what to do” [V4]
3. “This fear of mine depended on a series of factors (among which a fair amount of cliché): the main one was my shyness because I could not stand up to those with bullying attitudes and I was afraid that some child, maybe a bit more peculiar, could have annoyed me; the second reason was the fact that I was young […]. To put it simply, I was afraid I wasn’t “enough.” This year, however, went by so fast and little by little I acquired more awareness of myself and others” [V8]

In these extracts, the emotional load associated with the beginning of the experience is evident. Subjectivity was shaped using *embrayage* strategies aimed at conveying this sense of personal involvement (e.g., through contiguity markers such as “this” and “here” aimed at discursively shaping the “here and now” dimension of talk).

A second interesting cue in diatextual analysis was referred to as the A in the SAM model: Argumentation. The analysis of the argumentative texture of the discursive data collected helped to further investigate the strategies through which participants supported their positions. Even because of the special task requested to participants, diary pages were rich in narrative markers: participants told their personal stories giving several details regarding the description of the context, specific details about the protagonists, and the facts, thus revealing a profound sense of emotional involvement.

4. “At first, the three of us went to (visit) this old lady, Concetta. A woman aged 93! She had a puny body and her hands were a bit crooked, as she was used to work hard in the countryside. On her legs there was a light blue shawl, she had a scarf on her head and a little stove to keep herself warm. She looked at us with fearing eyes, but in the meanwhile, those eyes conveyed an infinite tenderness to us. We introduced ourselves and started to talk with her and his son. When it was time to say goodbye, we gave a kiss on her cheek. She held our hands and said: “I will see you tomorrow, right?” [V19]

The emotional tone of the narratives was also present in the use of direct discourse that participants used to describe facts and to recall specific episodes making them more vivid. This is a strategy mostly aimed at involving the reader in the scene, conveying participants’ emotional meaning attached to this experience.

5. “Every week she was punctual in her call:
  – “Hello Gina, tell me, doctor or shopping?”
  – “Hello *beddhra* [my beauty in local dialect], no I called you for the bread”
  – “Alright Gina, around 9.30 I’ll be there!”
  – “That’s fine, goodbye and thank you *fijia* [my child]”
  For me, Gina was ‘Lady Thank you’” [V19]
6. I got into a house; I saw a woman with a vitreous gaze. She was impassive at all of my questions, disappointed at her son just because he got me into the house, she looked at me and at him then she asked me: “why?.” Why did you enter in my garden, why did you enter in my house, in my life? Just to be together, I answered, to spend some time with me, with us, to speak and have some fun, to talk about the old harvests of tobacco, olives, corn…” [V23]

The M of Modality in the SAM model was mostly shaped through metaphors, a very powerful rhetorical device used to shape the volunteer/volunteering relationship. As previously affirmed, the beginning of the experience was not easy for many participants. To figure out this perception some used the image of the “bombing” to evoke a feeling of great disorientation in which they found themselves in the very first days.

7. The first days of Civil Service were a “bombardment” of news, timetables, places, and streets to remember, starting from the fastest way that go from the railway station to school […] I was always on the run especially because of the traditional delays of the *Sud Est* trains) [V9]

Metaphor was also used to describe the importance of the Universal Civil Service for the whole personal and professional experience of participants. The image chosen was: “the precious piece of a puzzle.” This metaphor marked the fundamental role played by this experience, labeled as “irreplaceable,” for the human and professional growth of participants.

8. “A precious and irreplaceable piece to build the puzzle of my future self-development in an apparently egoistic society. I would define my experience in the Universal Civil Service in this way: it ended a couple of years ago, but it is still vivid inside me” [V14]

A most traditional metaphor used to describe the context of this experience was “the family” metaphor. Volunteering activities were defined as a “second family,” where values, routines, and practices are important to grow up and to understand what is meaningful and important.

9. “To me, Civil Service was not a boring or annoying experience […] the elderly were my second family, they taught me to cherish the value of memories and savor every single instant […] to leave aside our frenzy and chaotic everyday life […] they passed me a different way of life […] a more beautiful and spontaneous one” [V22]

This image of growth was reinforced through some poetic metaphors recalling the importance of experiencing things with our five sense in order to keep memories alive and to treasure them (e.g., the color of nature in blossom, the sounds of the birds, the softness of snow, and the smell of the fireplace).

10. “A big family under the name of Civil Service […] well, for me Civil Service has been, is and will be my big family, my garden ready to blossom its flowers in winter, hummingbirds that spread melody in autumn, a soft snow on the cheeks of spring, a fireplace that give off heat in a fresh summer evening” [V17]

The profound bond between volunteer and volunteer work was further remarked by the metaphor of the “door” and the “key”: Volunteers perceived the importance of this experience in their life and felt they were tools to change the world.

11. You know, this project is not just for old people […] this project can tear down the barriers that society forces on us […] this project is the door and we are the keys […] we are ready to open our eyes on the world” [V19]

In a similar vein, the experience of Civil Service and the relationships that have been generated through it were described as a “key to happiness,” namely as a way through which volunteers felt they have found themselves and their place in the world.

12. I would have never thought that an old man aged 94 – only on his documents – could have given me the key to happiness (…) I would have never thought that he could have given me the keys of the door of my garden (…) helping me to enter and to tenderly taste my life” [V19]

### Volunteering as a Vocational Process

Another significant positive consequence of volunteering remarked by the literature (Barton et al., 2017; Kim and Morgül, 2017) is that it could be an opportunity to understand one’s own inclinations, making more effective study or work choices in the future. In this light, volunteering has been proved to be strictly linked to employability development (Knepper et al., 2015; Păceșilă, 2015).

These assumptions were also confirmed by the analysis conducted on the corpus of data collected: volunteering experience was defined as an opportunity to reflect on one’s personal and professional prospects and to change the perspective. It is a process of transformation that through contact with the real world pushed young people to reconsider themselves and their priorities, making them more aware of future goals and career management strategies, increasing individuals’ self-esteem and confidence, thereby growing a desire to apply for better jobs.

13. “[This experience relates to] transformation because getting in contact with the reality of the workplace has upset my system of values and principles giving them a new order, thus making me more mature, more responsible, more helpful toward others. [V27].

From a diatextual point of view, this positive aspect of the experience was also developed through agency markers such as “I,” “my” and volitional markers (“I want,” “I can”) as in extracts 13 and 14, aiming at “situating” the enunciator with reference to the context of talk and at underlining the personal responsibility and awareness about this choice and its consequences.

Furthermore, metaphors contributed to underline a high-emotional involvement of participants while reconstructing the frame of this experience.

In extract 14 two meaningful images emerged. The first recalled the power of Civil Service as an experience of epiphany (“I opened my eyes”) that could reveal and shed light on the life of volunteers showed clearly what was mostly unknown even to them, in terms of vocations and professional. The second image was evoked by the word “defeat”: this term is usually used to refer to the contraposition between different points of view and claims, here was adopted to strengthen the enunciator’s positive appreciation of this experience. It is used as a moralization strategy, appealing to common sense and to widely shared social values, and is useful to further legitimize one’s own argumentation (Van Leewen, 1996).

14. “In the end, I can say that thanks to this experience because I have opened my eyes on the future, resuming my studies and being aware that I wanted to help those people who live any kind of distress, including children, because everyone deserves to know that there are people who work to defeat things that can be a problem”

Finally, the experience of voluntary work in the Universal Civil Service allowed some participants to reconsider their educational choices. In extract 15 the experience of volunteering enabled this girl to discover a professional field she never considered and to acquire useful knowledge and skills for future inclusion in that context. Diatextual analysis allowed to catch the argumentative texture of the narrative through the recurrence of a discursive network of logoi and antilogoi, aimed at explaining how the girl came to change her mind about her future. The dialog between her previous positions, namely the logoi (“I’ve never,” “I could not,” “I had another project,” “I wanted”) and her current discovery, which are the antilogoi (“instead,” “conversely to what you think”), was developed through an inner dialog between opposing stances and opinions about her future and consequently about the meaning attached to the experience of the Civil Service. The argumentative program emerging from this extract was constructed through the dialectic between the categories of “certain” (her beliefs and supposed vocations before the experience) vs. “uncertain” (the concrete experience of Civil Service). This interlocutory exchange allowed the volunteer to become aware of her real inclinations and desires and to take the responsibility to make courageous choices for her future.

15. I’ve never considered the idea of working in school; I could not see it as a possible working scenario, I had other projects for me: I wanted to graduate in Pharmacy, to become a researcher, because I’ve never seen myself in something different than a scientific role. Instead, the Universal Civil Service has turned things upside-down […] it left me with many doubts on my future professional career, even though I already was 27 years old and I thought to know what I wanted […] now it is 3 years that I have concluded my experience in that school and I still have vivid memories that return to me from time to time [V3].

### Volunteering as a Context for Employability Capital Development

As argued in the literature review, voluntary work could be a precious tool for professional development and consequently for enriching one’s own employability capital. The analysis of the narratives collected confirmed this evidence showing a surprising awareness of volunteers especially about the capital of soft skills they have learnt through this experience. Yet, this precious capital encompasses soft skills that were very similar to those pointed out by other contributions in the field and specifically related to volunteering (Ren and Du, 2014; Khasanzyanova, 2017). These skills are featured by personal resources (effectiveness, listening, adaptability, etc.), communication resources (knowing how to explain, communication with members and beneficiaries of the associations, etc.), interpersonal resources (sense of responsibility, teamwork, organizational skills, etc.), and value-driven behaviors (solidarity, passion, understanding, etc.).

Personal skills emerged as decisive to manage the core activities of the Civil Service. In extracts 16 and 17, volunteers declared that the Civil Service was a ground where they acquired fundamental personal skills: “humility,” “respect,” and “spirit of hospitality” were some of the personal skills learned during volunteer work and represented the reason why volunteers felt grateful to this experience. In extract 17, the volunteer referred that the Civil Service experience was useful above all to increase self-awareness, to face one’s fears, to overcome one’s own limits, to take on responsibilities. Later, these skills were recognized as fundamental to access to the labor market and confirm the importance of this experience for increasing the potential future employment of volunteers.

16. “I believe that thanks to this experience I have acquired humility and respect toward other people’s suffering, spirit of hospitality and listening abilities, respect toward diversity and other people’s time needs” [V3]
17. “I cannot deny that this experience has helped me a lot; it has helped me to grow up because I soon felt the responsibility toward those children, the responsibility to carry on my tasks […] I dealt with many of my fears and I overcame my limits.” [V11]

The second category of soft skill recognized as core skills acquired and developed during this experience was communication skills. Traditionally, communication skills relate to the ability to manage a discussion, to build a social network, and are recognized as particularly salient in any sphere of life as they can be acquired especially in non-formal and informal contexts.

In extracts 18 and 19, two fundamental dimensions emerged: the ability to establish effective “circles of communication” and the ability to capitalize “a relational network.” Both are crucial skills to successfully enter the labor market and to develop one’s own employability.

18. “For me this experience has been useful to learn how to establish an effective circle of communication among the family, the school, the territory, the public services, and the parochial context” [V6]
19. “This experience has allowed me to meet many people with different professional skills and has helped me a lot to build a relational network that has been important for me both to effectively carry out the work that was required to do but I know that it will be useful even after this experiencer” [V7]

Strictly linked to communication skills, volunteers identified interpersonal skills as a precious capital acquired in Civil Service. These skills focused on the ability to interact, collaborate with, and have good symmetrical and asymmetrical relationships with others (Kechagias, 2011). This category of skills includes the ability to work in a team, to adapt to situations, to negotiate, to organize one’s own and others’ work, etc.

The most significant interpersonal skills that volunteers told they learned were listed in the extracts below and showed a great awareness about their resources and the ability to transform every single moment of this experience into an opportunity for professional growth. In extract 21, it was interesting to note how the volunteer reported that the skills learned were fundamental “for my everyday life,” suggesting their transversal nature as well as the ability to treasure them and to transfer them to a future different working context.

20. “Attention toward the others, team collaboration, organizational skills, responsibility, resistance to stressful or sensitive situations: these are only some of the skills I have acquired in this experience” [V14]
21. “Personally, this experience has improved aspects of my everyday life, like dealing with different situations and learning to collaborate and plan activities with other people” [V20]

Finally, as for the category of soft skills labeled “various skills” by Ren and Du (2014) and mostly related to value-driven behaviors, volunteers referred to values, passion, dedication to people in difficulty, assistance to the elderly, to deviant children. In extract 22, voluntary work experience was described as a context where participants had occasion to grow up, to improve, to develop passion and dedication for what they were doing. This was the key to start changing the self, also fostered by the acknowledgement of an inspiring relational context, full of people who like in a family believe in the same values and attain the same objectives.

22. “The Civil Service experience is passion, dedication, desire to learn and challenge yourself, also in relation to others […] it is being part of a family that shares your same ideals and that changes you and helps you to grow up” [V15]

This process of personal change initiated through the experience of volunteering was further amplified in extract 23, where the volunteer pointed out three keywords, he/she learnt, and jealously kept as “secrets.” The use of this metaphor contributed to discursively construct some basic features of this experience, namely rarity and uniqueness. The secrets he/she learnt were so precious that the young volunteer said he/she wanted to keep and to store them in the luggage because they surely will be used along the way during the journey.

23. “Love, empathy, friendship: these are the secrets that I brought with me in this journey called Civil Service, and that I will always take with me in the luggage of life” [V19]

## Discussion and Conclusions

The main aim of the study was to investigate the participation of the Universal Civil Service as an experience of informal and non-formal workplace learning useful to develop an employability capital.

The focus on this special context was justified by the assumption that the asset of knowledge and soft skills acquired through this opportunity could be considered as an intangible and strategic capital both for individuals as well as for organizations (Buonomo et al., 2020). Therefore, the Civil Service is an experience that students and/or young graduates often chose before concretely entering the labor market; the study argued for the crucial role it could take as a context of vocational guidance, being useful to become more aware of the personal resources that will be strategic for future career management (Manuti, 2019). However, given that informal and non-formal learning are mainly tacit, implicit, and featured by unstructured learning occasions, a conscious elaboration of this experience and the capitalization and transformation of learning into concrete and spendable professional skills are fundamental to further develop volunteers’ career identity. To this purpose, narratives were used as a tool to collect data, because it was supposed that they could facilitate a “longitudinal” (Fugate et al., 2004, p. 20) sense-making process about past, present, and future career management trajectories.

In light of these reflections, results coming from the present study provided precious insights both for theory development as well as for the improvement of professional practices, connected with young volunteers’ training and vocational guidance in this non-profit sector.

At a theoretical level, data analysis contributed to investigate the relationship between voluntary work and employability development, voicing the point of view of volunteers. Yet, although being a qualitative study, therefore providing a partial although intense account of experience in a specific geographical and cultural context, the narrative data collected confirmed the centrality of three crucial aspects: the non-formal and informal dimensions implied in workplace learning developed during voluntary work, the transferable potential of the softs skills acquired, the usefulness of these contexts of experience for developing awareness about one’s own employability capital.

The Universal Civil Service was described as a natural context for non-formal and informal learning, where young people had multiple occasions to learn by doing and to learn through meaningful relationships with others. Yet, it is through these positive encounters during service work that volunteers had opportunities to discover something more about themselves, about their values, their inclinations, their abilities, and their vocations, thus acquiring highly transferable soft skills, that are potentially useful for their professional future. In this perspective, the Universal Civil Service became a concrete experience of personal vocational guidance, where young people discovered “who they were and what they wanted to be” (Fugate et al., 2004, p. 20) in their future. It was thanks to this experience that volunteers consciously nourished a capital of personal resources and of social networks that could be transformed into a precious although intangible asset of employability for their future (Duvekot et al., 2007; Imperial et al., 2007; Waldner and Hunter, 2008; Conway et al., 2009; Reason and Hemer, 2010; Finley, 2011). A further contribution to theory development was given by the methodological perspective adopted by the study. The focus on volunteers’ narratives and the adoption of diatextual analysis as a lens to go through them allowed them to go over a simple account of contents, revealing how affective and cognitive dimensions of volunteers’ career identity weaved together in complex narrative textures.

From a practical point of view, results from the study highlighted the potential that such experience might represent for the communities and for the contexts hosting volunteers. Accordingly, as already underlined earlier, this experience potentially provides multiple occasions to develop highly transferable knowledge and skills both for groups and individuals. In this light, what could be an employability capital for the young people involved, could also be a strategic and intangible asset for the organizations hosting volunteers.

Yet, research findings suggested that the development of a strategic plan for the socialization of newcomers and for formal and informal training of volunteers could help managers and tutors to most fruitfully manage this special kind of human resources, who although making a voluntary experience might represent a precious, even if transitory, human capital, making the difference in terms of performance quality (Buonomo et al., 2020).

Likewise, special attention should be paid to the process of volunteers’ recruitment and to the training of tutors.

In the first case, narratives about the experience of participation in the Universal Civil Service highlighted the importance of the fit between volunteers’ personal values and those conveyed by the contexts where they served, both from an individual perspective, because it helped volunteers to recognize future career goals and motivations, but also from an organizational perspective, since the individual perception of a person/organization fit contributed to strengthening positive organizational behaviors, such as commitment, identification, and engagement, and consequently might impact on performance.

Finally, the study gave some useful suggestions also for the training of the tutors responsible for the transfer of skills, abilities, work attitudes, and values that should inspire behavioral patterns and working activities according to a well-defined organizational culture. The metaphor of the family recurrent in volunteers’ narratives was informative in this sense because it showed the importance of sharing the same vision and mission in order to adjust also volunteers’ attitudes and behaviors according to the same cultural model.

Despite these positive aspects, some limitations could also be highlighted. First, the study focused on a specific volunteering program, such as the Universal Civil Service, which has specific characteristics, and which inevitably excludes many other experiences that could offer different opportunities for the acquisition of skills and the growth of volunteers. However, if, on the one hand, this evidence granted the opportunity to investigate the positive impact of the program on the development of crucial skills useful for the transition of volunteers to the labor market, on the other hand, it cannot be exhaustive of the complexity of volunteering as a wider context of informal and non-formal learning.

In this regard, future research could adopt a longitudinal qualitative design to investigate volunteers’ transfer of learning from this informal context to an actual professional one, by collecting similar in-depth interviews across time and examining if and to what extent the experience of voluntary work would have intentionally become part of the employability capital of young volunteers.

Moreover, different contexts of voluntary work could be explored in order to trace differences and peculiarities among them, focusing on the conditions that could foster the development of an employability capital (e.g., the role of the tutor, the organizational culture, the organization of tasks and responsibility, etc.).

A second limitation was the limited number of participants involved that could provide a partial and “cross-sectional” view of the phenomenon. Certainly, the selection of a small group of volunteers responded to the specific criteria of qualitative analysis (e.g., to be intensive more than extensive in the analysis and in interpreting data), but at the same time, it failed to catch the extreme complexity of the object of research and cannot lead to generalizable results. Therefore, future research could adopt a multi-method design, triangulating qualitative and quantitative methodologies and capitalizing on potentialities of both in the textual analysis (Cortini and Tria, 2014).

In conclusion, despite these evident limitations inherent to qualitative research, the present study contributed to shed lights on the peculiarities of a special kind of voluntary work, on its challenges and opportunities both for individuals and organizations, paving the way for the future development of specific HRM strategies useful to develop, keep, nourish, and manage the intangible human, social, and employability capital of volunteers.

## Data Availability Statement

The raw data supporting the conclusions of this article will be made available by the authors, without undue reservation.

## Ethics Statement

The studies involving human participants were reviewed and approved by Ethics committee Department of Education, Psychology and communication – University of Bari (Ethics reference code: ET-20-11). The participants provided their written informed consent to participate in this study.

## Author Contributions

AM and MG jointly conceived and developed the research project. AM wrote the introduction and the literature review. MG wrote the data analysis sections. Discussion and Conclusions were written by both authors. All authors contributed to the article and approved the submitted version.

### Conflict of Interest

The authors declare that the research was conducted in the absence of any commercial or financial relationships that could be construed as a potential conflict of interest.

## Footnotes

^1^For privacy reasons participants were coded with a progressive number. Letter V stands for Volunteer.
